# MHD Forced Convective Laminar Boundary Layer Flow from a Convectively Heated Moving Vertical Plate with Radiation and Transpiration Effect

**DOI:** 10.1371/journal.pone.0062664

**Published:** 2013-05-31

**Authors:** Md. Jashim Uddin, Waqar A. Khan, A. I. Md. Ismail

**Affiliations:** 1 American International University-Bangladesh, Banani, Dhaka, Bangladesh; 2 Department of Engineering Sciences, PN Engineering College, National University of Sciences and Technology, Karachi, Pakistan; 3 School of Mathematical Sciences, Universiti Sains Malaysia, Penang, Malaysia; University of Adelaide, Australia

## Abstract

A two-dimensional steady forced convective flow of a Newtonian fluid past a convectively heated permeable vertically moving plate in the presence of a variable magnetic field and radiation effect has been investigated numerically. The plate moves either in assisting or opposing direction to the free stream. The plate and free stream velocities are considered to be proportional to 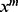 whilst the magnetic field and mass transfer velocity are taken to be proportional to 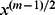
where 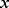
is the distance along the plate from the leading edge of the plate. Instead of using existing similarity transformations, we use a linear group of transformations to transform the governing equations into similarity equations with relevant boundary conditions. Numerical solutions of the similarity equations are presented to show the effects of the controlling parameters on the dimensionless velocity, temperature and concentration profiles as well as on the friction factor, rate of heat and mass transfer. It is found that the rate of heat transfer elevates with the mass transfer velocity, convective heat transfer, Prandtl number, velocity ratio and the magnetic field parameters. It is also found that the rate of mass transfer enhances with the mass transfer velocity, velocity ratio, power law index and the Schmidt number, whilst it suppresses with the magnetic field parameter. Our results are compared with the results existing in the open literature. The comparisons are satisfactory.

## Introduction

Heat and mass transfer is an important flow transport process which is driven by a combination of both solute and thermal diffusion effects [1]. This type of problem is encountered in fields such as geology, oceanography and astrophysics as well as practical applications such as the design of energy efficient building components, control of the pollutant spread in groundwater and compact heat exchangers [2]–[3]. Makinde [4] studied similarity solution of MHD flow with heat and mass transfer over a moving vertical plate. He included magnetic field and thermal convective surface boundary condition in his study. In another paper, the same author [5] presented similarity solutions of the hydromagnetic mixed convective flow along a vertical plate in a porous medium which includes radiation and the heat generation effect. Magnetohydrodynamic flows have received the attention of many investigators because of their many engineering applications. In metallurgy, for example, some processes require the cooling of continuous strips by drawing them through an electrically conducting fluid which is subject to a magnetic field. This allows the cooling to be controlled and final product with the requisite properties obtained [6]. Many researchers have investigated MHD flows past various geometries in porous as well as clear media. For example, Pal and Mondal [7] studied the flow and heat transfer effects of an electrically conducting fluids such as liquid metals; water mixed with a little acid and other equivalent substances in the presence of a magnetic field. Herdrich et al. [8] pointed out possible applications of magnetically controlled plasmas in space technology. The similarity solutions for MHD flow with heat transfer over a wedge considering variable viscosity and thermal conductivities effects was investigated by Seddeek et al. [9]. Alam et al. [10] discussed the effects of suction and thermophoresis on steady MHD combined free-forced convective heat and mass transfer flow over an inclined radiative plate. Rahman and Salauddin [11] dealt with the effects of variable electric conductivity and viscosity on hydromagnetic heat and mass transfer flow. Hendi and Hussain [12] presented solution of MHD Falkner-Skan flow over a porous surface by homotopy analysis method. Bararnia et al. [13] obtained analytical solutions of the Falkner–Skan equations with heat transfer using the homotopy perturbation method.

The forced convective flow past a moving plate in a moving fluid has numerous application in engineering including the cooling of polymer sheets and plates and conveyor belts. Thermal transport due to a continuously moving plate through quiescent or moving fluid find applications in processes such as plastic extrusion and continuous casting. Therefore, the knowledge of the flow and thermal fields at the vicinity of the plate is required for ensuring, the quality of the manufactured products [14]. Klemp and Acrivos [15] presented a method for integrating the boundary layer equations through a region of reverse flow. They applied the method to uniform flow past a parallel flat plate. Similar problems have also been studied by Weidman [16], Ishak et al. [17] amongst others.

Heat and mass transfer due to forced convection occurs in chemical process, biochemical process and engineering. Ali [18] investigated the effect of temperature dependent viscosity on laminar mixed convective flow and heat transfer for a continuously moving vertical isothermal surface and obtained local similarity solutions. According to previous studies, the forced convection flow over a moving plate has engineering applications such as liquid films in condensation process and in aerodynamics. Similarity solutions about moving plate were investigated by many authors. Boundary layer flow along a moving plate parallel to a moving stream was studied by Ishak et al. [19]. A similar problem also was studied by Ishak [20] in 2010. Magyari [21] studied the moving plate thermometer with uniform velocity using the Merkin transformation method. Hoernel [22] reported the similarity solutions for steady MHD flow near the forward stagnation point of two-dimensional moving axisymmetric bodies.

The flow of an electrically conducting fluid with thermal radiation occurs for example in electrical power generation and solar power technology. The radiative heat mass transfer also occurs in a variety of geophysical and engineering applications such as migration of moisture through air contained in fibrous insulations, nuclear reactors, nuclear waste disposal and others [23]. The temperature as well as heat transfer may affect radiation, at high operating temperature. Radiation plays an important role in controlling heat transfer in certain processes. Its effects on boundary layer flow with or without magnetic field have been investigated by amongst others: Duwairi and Duwairi [24], Cortell [25], Bataller [26], El-Kabeir et al. [27], Ishak et al. [28], Bakier [29], Cortell [30] etc. All of the above mentioned researchers limited their investigation to either isothermal or isoflux thermal boundary conditions. It seems appropriate to us to generalize the previous investigations to a thermal convective boundary condition instead of an isothermal or isoflux boundary condition.

The present study attempts to pinpoint the combined effect of the transpiration velocity and the radiation on the magnetohydrodynamic forced convective flow of a viscous incompressible Newtonian fluid past a convectively heated moving vertical plate in the moving free stream which have by far not been elucidated in the literature. In an effort to achieve this goal, we shall use a linear group of transformation to develop the similarity transformations (not using the existing transformations) and hence the corresponding similarity representation of the governing equations, before being solved numerically by the Runge-Kutta-Fehlberg fourth-fifth order numerical method. The effect of the controlling parameters the velocity ratio, mass transpiration, radiation, magnetic field, power law index, convective heat transfer on the flow, heat and the mass transfer characteristics are investigated and analyzed.

## Mathematical Modeling of the Problem

**Figure 1:**
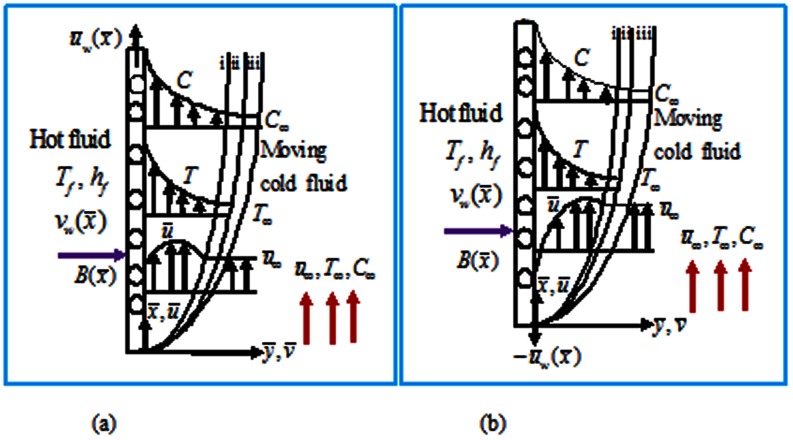
Flow configuration and coordinate system when (a) plate and free stream move in the like direction (b) plate and free stream move in the unlike direction.

Consider a 2-D MHD forced convective flow along a moving permeable radiating convectively heated vertical flat plate as shown in **Fig. 1**. (i, ii, iii respectively represent velocity, temperature and concentration boundary layers). It is assumed that an electrically conducting cold fluid having an electric conductivity 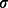 in the presence of a magnetic field of variable strength 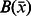 is flowing along the flat plate. It is considered that the plate is stretched with a power law velocity and the magnetic field is normal to the plate. Further, the left surface of the plate is assumed to be heated by convection from a hot fluid of temperature 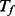 and this generates a variable heat transfer coefficient 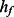. The temperature and concentration at the wall are 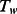
and 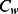. Ambient temperature and concentration are 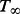 and 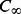. Fluid properties are assumed to be invariant. The particle coagulation, magnetic Reynolds number, electric field due to polarization of charges and Hall effects are assumed to be negligible. Under these assumptions the governing transport equations in dimensional form are [22], [31].
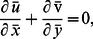
(1)

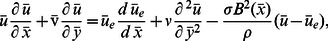
(2)

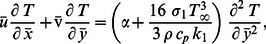
(3)

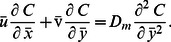
(4)subject to the boundary conditions
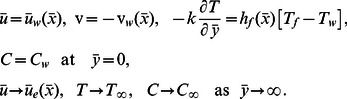
(5)Here 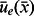: the potential velocity, 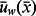: the velocity of the plate, 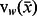: the velocity normal to the plate with 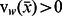 for injection (blowing), 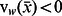 for suction and 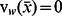 corresponds to an impermeable plate. As usual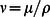: the kinematic coefficient of viscosity, 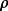: the density of the fluid, 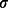: the electric conductivity, 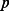: the pressure, 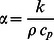: the thermal diffusivity, 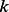: the thermal conductivity, 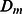: the mass diffusivity, 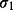: the Stefan-Boltzmann constant, 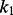: the Rosseland mean absorption coefficient.

### Nondimensionalization

Introducing the following dimensionless boundary layer variables
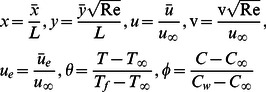
(6)with 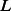 being the characteristic length, 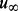
is the reference velocity and 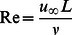 is the Reynolds number. We assume the following
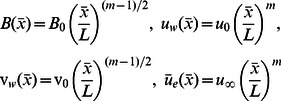
(7)where 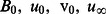
are constants [22]. Here *m* (not necessarily an integer) is the power law exponent, 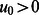 or 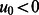 mean the plate is moving along positive or along negative direction of 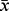
- axis and 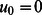 represent a stationary plate. Introducing the stream function 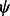 so as to reduce the number of the dependent variables as well as the number of the equations. 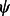 is defined by
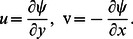
(8)into Eqs. (2–5), we have,
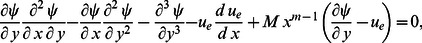
(9)

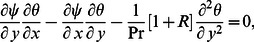
(10)

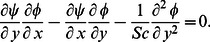
(11)The boundary conditions are
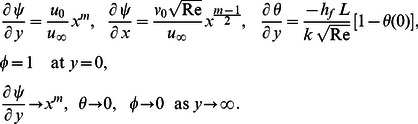
(12)Here 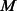 is the magnetic field parameter, 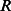 is the radiation parameter, 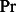
is the Prandtl number, 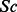 is the Schmidt number, which are defined as
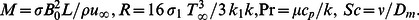
(13)


### Scaling Symmetries

The independent and dependent variables are scaled as
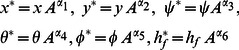
(14)where 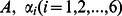
are constant [32]–[34]. Values of 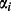 are sought such that the form of the Eqs. (9)–(12) remain invariant under the transformation. Substituting our new variables in Eqs. (9)–(12), equating powers of 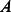
(to ensure the invariance of this equation under this group of transformations), we have,
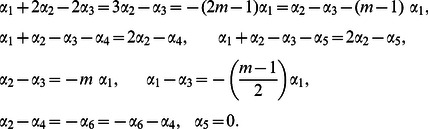
(15)Solving (15), we have
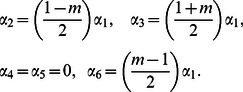
(16)We then seek the functions having the same form before and after the transformation, (i.e., absolute invariants).

It is seen from Eqs. (14) and (16) that
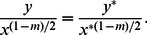
(17)This combination of the variables is hence an invariant under this group of transformation and so is an absolute invariant. We denote this functional relationshp by
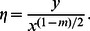
(18)Using the same techniques, other absolute invariants are
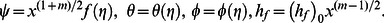
(19)Here 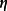 is the similarity independent variable, 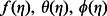 are similarity dependent variables and 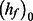 is the constant heat transfer coefficient.

Substituting Eqs. (18) and (19) into Eqs. (9)–(12), lead to the following ordinary differential equations
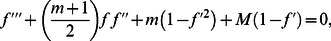
(20)

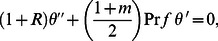
(21)

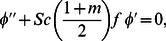
(22)subject to the boundary conditions
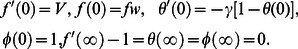
(23)where 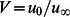 (constant) is the velocity ratio parameter with 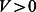 means that motion of the plate is in the same direction as the free stream velocity and 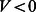 means the plate is moving in the opposite direction of the free stream and 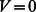 stands for static plate. The case 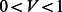 means the speed of the plate is less than that of the free stream fluid and 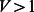 mean the speed is greater. 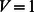 is the case when the plate and the fluid move with the same velocity. Here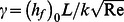 is the convective heat transfer parameter and 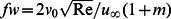 is the suction/injection parameter, primes denote ordinary differentiation with respect to 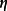. We have successfully transformed a set of partial differential equations into a set of ordinary differential equations with relevant boundary conditions. The solutions of the transformed equations are much easier than the original equations.

### Physical Quantities

The quantities we are interested in are the Skin friction factor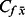 (wall shear stress), the local Nusselt number (heat transfer rates) 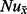 and the local Sherwood number (mass transfer rates) 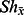 respectively. They can be derived from the following definitions:
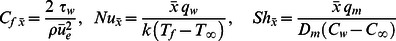
(24)where 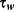 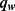, 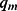 are the wall shear stress, the wall heat and the wall mass fluxes, and are defined as
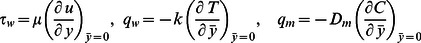
(25)By substituting from Eqs. (6), (18) and (19) into Eq. (25), we get
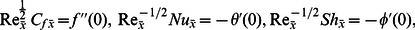
(26)where 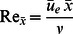 is the local Reynolds number.

### Special Cases

It is interesting to note that in case of the purely hydrodynamic boundary layer (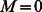), if (i) the full stream velocity is constant 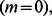 (ii) the plate is impermeable 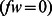, (iii) the plate is stationary 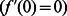, (iv) radiation is absent (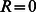), our problem reduces to Aziz [35]. Further, in case of purelyhydrodynamic boundary layer (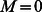), if (i) the full stream velocity is constant 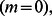 (ii) the plate is permeable 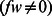, (iii) the plate is stationary, (iv) radiation is not present (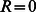), our problem reduces to Ishak [28].

It is further interesting to note that our problem reduces to Cebeci and Bradshaw [36] in case of purely hydrodynamic boundary layer (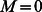) flow past a stationary 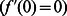 isothermal (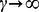), non-radiating (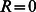) plate. In this case, Eq. (22) has no meaning.

For non-radiating plate, with the following minor modifications
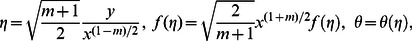
(27)
Eqs (20) and (21) become
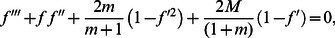
(28)

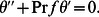
(29)subject to the boundary conditions
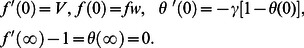
(30)In the absence of an external magnetic field (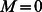), Eqs. (28) and (29) are identical to Suhil and Al-Nimr [37].

In the absence of energy Eq. (29), we have,
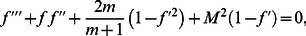
(31)subject to the boundary conditions
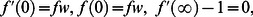
(32)where 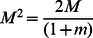. Note that Eq. (31) is the MHD Falkner-Skan equation which has been recently investigated by Hendi and Hussain [12].

For stationary impermeable isothermal plate 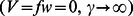 and for purely hydrodynamic flow 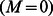, Eqs. (28)–(30) become
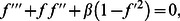
(33)

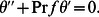
(34)subject to the boundary conditions
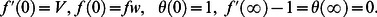
(35)The parameters 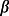
and 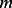 are related through the expression 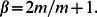 The parameter 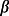 is the Hartee pressure gradient parameter.

It is further worth citing that Eqs. (33)–(35) are similar to Eqs. (16), (19), (17) and (20) of the most recent paper of Bararnia et al. [13].

### Numerical Solution

Similarity Eqs. (20)–(22) with boundary conditions (23) were solved numerically using a fourth-fifth order Runge-Kutta-Fehlberg numerical method. This method has been used in several convective heat and mass transfer problems. The step size 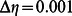 was used while obtaining numerical solution with 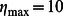 and the accuacy to the sixth decimal place was found sufficient for convergence. The asymptotic boundary conditions given by Eq. (23) were replaced by using a value of 15 for the similarity variable 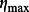 as follows.
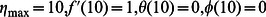
(36)The choice of 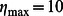 ensured that all numerical solutions approached the asymptotic values correctly. This is an important point that we should keep in mind while solving boundary layer problems.

## Results and Discussion

**Figure 2:**
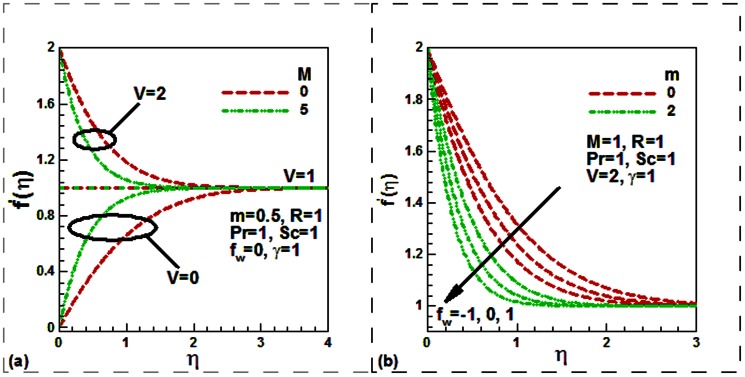
Effect of (a) the velocity ratio and magnetic field parameters and (b) the power law index and mass transfer velocity parameters on the dimensionless velocity.

**Figure 3:**
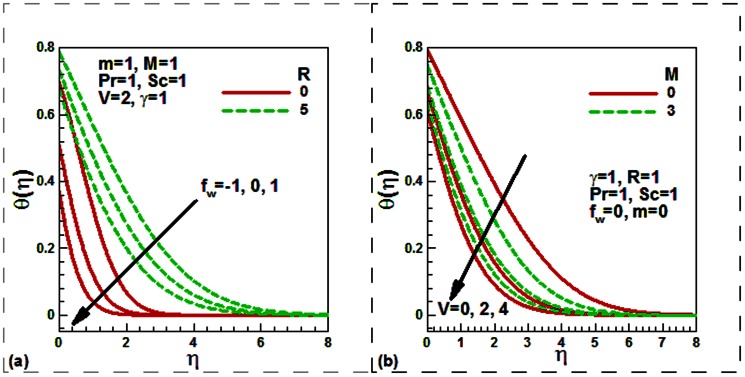
Effect of (a) the radiation and mass transfer velocity parameters and (b) the magnetic field and velocity ratio parameters on the dimensionless temperature.

**Figure 4:**
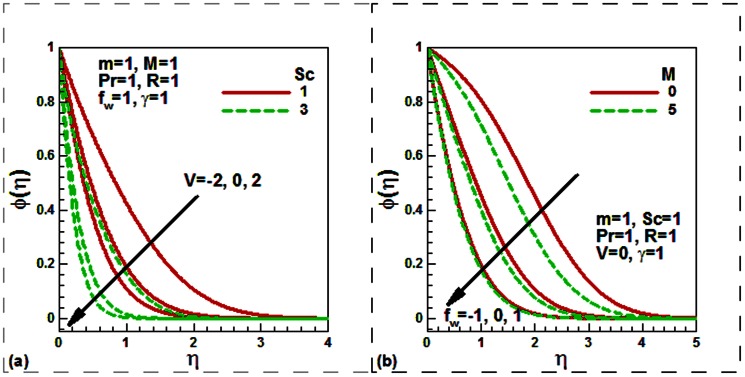
Effect of (a) the velocity ratio and Schmidt number and (b) the magnetic field and mass transfer parameters on the dimensionless concentration.

**Figure 5:**
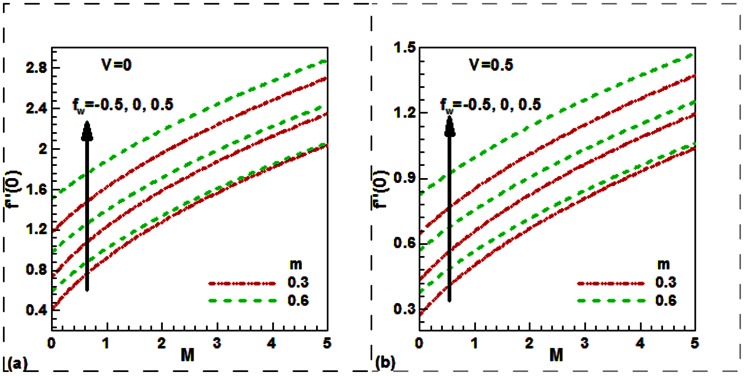
Variation of the dimensionless friction factor with magnetic field parameter, power law index parameter and suction/injection parameter for (a) stationary plate, (b) moving plate moves along positive axial axis.

**Figure 6:**
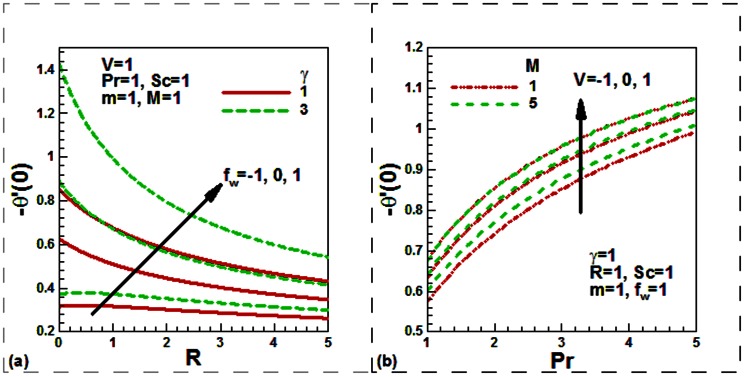
Variation of the dimensionless local heat transfer rates (a) with the radiation, convective and suction/injection parameters and (b) with the Prandtl number, velocity ratio and magnetic field parameters.

**Figure 7:**
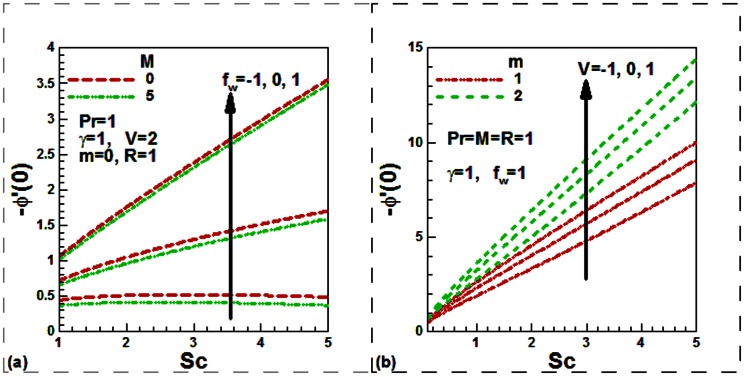
Variation of the dimensionless local mass transfer rates with (a) the Schmidt numbers, magnetic field and suction/injection parameters and (b) the Schmidt numbers, velocity ratio and power law index parameters.

Our numerical results are compared with Aziz [35] in Table 1, Jafar et al. [38] in Table 2, Cebeci and Bradshaw [36] and Yih [40] in Table 3, Hendi and Hossain [12] and Abbasbandy and Hayat [39] in Table 4, and Barania et al. [13] and Rajagopal et al. [42] in Table 5, Hendi and Hossain [12] and White [41] in Table 6 to justify the accuracy of the method used. The comparisons are satisfactory. Figures 2, 3, 4, 5, 6, 7 exhibit the emerging parameters effect on the flow and heat and the mass transfer characteristic.

**Table 1:** Comparison of results of 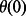 and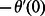 for different values of 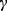 when 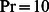.

	Aziz [35]		Present results	
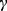	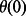	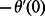	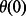	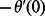
0.05	0.0643	0.0468	0.06426	0.04679
0.10	0.1208	0.0879	0.12075	0.08793
0.20	0.2155	0.1569	0.21548	0.15690
0.40	0.3546	0.2582	0.35457	0.25818
0.60	0.4518	0.3289	0.45176	0.32895
0.80	0.5235	0.3812	0.52351	0.38119
1.00	0.5787	0.4213	0.57866	0.42134
5.00	0.8729	0.6356	0.87288	0.63558
10.00	0.9321	0.6787	0.93213	0.67872
20.00	0.9649	0.7026	0.96487	0.70256

**Table 2:** Comparison of skin friction 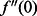 for various combinations of 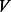, 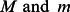.

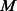	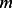	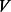	Present results	Jafar et al. [38]
1	−0.7	−1.2465	1.785854	1.785854
1	−0.7	−0.3	0.626503	0.6261
1	−0.7	0.5	−0.036115	−0.0361
1	−0.7	1.1	−0.048819	−0.0488
1	−0.6	−1.2465	1.831134	1.8311
1	−0.6	−0.3	0.754875	0.7549
1	−0.6	0.5	0.147122	0.1471
1	−0.6	1.1	0.008662	1.6444
2	−0.7	−1.2465	2.8552230	2.8552
2	−0.7	−0.3	1.4340566	1.4341
2	−0.7	0.5	0.4700876	0.4701
2	−0.7	1.1	−0.0799084	−0.0799
2	−0.6	−1.2465	2.8886778	2.8887
2	−0.6	−0.3	1.5013537	1.5014
2	−0.6	0.5	0.5167744	0.5168
2	−0.6	1.1	−0.093447	−0.0935

**Table 3:** Comparison of skin friction 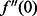 with Cebeci and Bradshaw [36] and Yih [40].

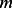	Present results (RKF45)	Cebeci and Bradshaw [36] (Finite difference)	Yih [40] (Finite difference)
−0.05	0.2134838	0.21351	0.213484
0.0	0.3320574	0.33206	0.332057
1/3	0.7574476	0.75745	0.757448
1.0	1.2325876	1.23259	1.232588

**Table 4:** Comparison of skin friction 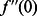 with Hendi and Hosssain [12] and Abbasbandy and Hayat [39] for 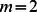.

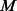	Present results (RKF45)	Hendi and Hosssain [12] (HPM-Pade)	Abbasbandy and Hayat [39] (Finite difference)
1	1.7194657	1.719465	1.719465
2	2.4394989	2.439498	2.439498
5	5.1909598	5.190959	5.190959
10	10.0967757	10.096775	10.096775
50	50.0194408	50.019440	50.019440

**Table 5:** Comparison of skin friction 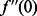 with Barania et al. [12] and Rjagopal [42].

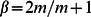	Present results (RKF45)	Barania et al. [13] (HPM-Pade)	Rjagopal [42] (Finite differences)
0	0.469600	0.46964	0.4696
0.5	0.531130	0.53119	0.5311
0.1	0.587035	0.58716	0.5870
0.2	0.686708	0.68672	0.6867
0.3	0.774755	0.77475	0.7747
0.4	0.854421	0.85442	0.8544
0.6	0.995836	0.99589	0.9958
0.7	1.059808	1.05985	-
0.8	1.120268	1.12020	1.1202
0.9	1.177728	1.17699	-
1	1.232587	1.23150	1.2325
1.2	1.335721	1.33559	1.3357
1.6	1.521514	1.52141	1.5215
2	1.687218	1.68462	-

**Table 6:** Comparisons of Nusselt numbers with Barania et al. [13] and White [41].

	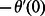					
	Present results (RKF45)		Barania et al. [12]HPM-Pade)		White [41] (Finite difference)	
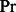	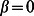	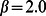	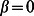	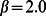	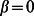	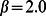
0.1	0.19885	0.22654	0.19765	0.22622	0.1980	0.2260
0.3	0.30371	0.36681	0.30401	0.36617	0.3037	0.3668
0.6	0.39167	0.49130	0.39174	0.49149	0.3916	0.4913
0.72	0.41809	0.52960	0.41813	0.52932	0.4178	0.5292
1	0.46959	0.60520	0.46964	0.6055	0.4696	0.6052
2	0.597233	0.79599	0.59726	0.79522	0.5972	0.7959
3	0.68596	0.93035	0.68589	0.93082	0.6859	0.9303
6	0.86728	1.20692	0.86723	1.20631	0.8672	1.2069
10	1.02975	1.45575	1.03003	1.45520	1.0297	1.4557
30	1.48732	2.15773	1.48741	2.16013	1.4873	2.15773
60	1.87459	2.75196	1.87469	2.75310	1.8746	2.7520
100	2.22291	3.28625	2.22289	3.28674	2.2229	3.2863
400	3.52923	5.28901	3.52917	5.28961	3.5292	5.2890

Fig. 2 (a) depicts the effect of the magnetic field and velocity ratio parameters whilst Fig. 2 (b) exhibits the effect of the power law index and the transpiration velocity parameters on the dimensionless velocity. The magnetic field leads to decrease the velocity and then the boundary layer thickness shrinks when the plate moves faster than the free stream in the same direction 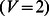. This is due to the fact that applied magnetic force act like a drag force which reduces the velocity. It is interesting to note that in the case of the stationary plate 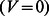 the magnetic field causes to elevate not retard the velocity (Fig. 2 (a)) (as in classical MHD boundary layer flow. Physically this is because for stationary plate in the moving free stream, application of the magnetic field which is moving with the free stream has the tendency to induce the motive force which induces the motion of the fluid and lessens the boundary layer thickness. Clearly, an increase in the velocity ratio leads to an increase in the dimensionless velocity for both electrically non-conducting flow 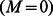
and electrically conducting flow 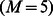 solid plate in the uniform free stream.

It is found from Fig. 2 (b) that the velocity suppresses with the rising of the nonlinear power law parameter 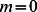 for both permeable 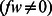 and solid 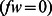 convectively heated plates. This figure is drawn by taking 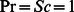
which means that the momentum, heat and mass diffuse at the same rate. This result is consistent with the know results in the literature. It is also found that the velocity enhances with the suction and suppresses with the injection for both constant free stream 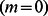 and quadratically varying free stream 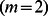. An illustration for this trend is that the fluid is brought nearer to the plate in the case of suction 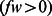, and the reverse behavior is noticed for the case of injection 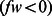.Note that this figure is drawn for the plate which moves in the same direction of free stream with velocity faster than free stream.

The influences of the radiation, mass transfer velocity, magnetic filed and the velocity ratio parameters on the dimensionless temperature profiles are shown in Fig. 3. The temperature reduces with the injection and rises with the suction both for the radiating and non-radiating moving plate. The thermal radiation is found to increase the temperature both for permeable and solid plate (Fig. 3 (a)). This is because the inclusion of the thermal radiation increases the thermal diffusivity (Eq. 2) which then increases the temperature and hence reduces the heat transfer rate. This is what we observe from Fig. 6a. It is noticed from Fig. 3 (b) that the velocity ratio leads to reduce the dimensionless temperature for both the hydrodynamic and magnetohydrodynamic boundary layer flows. This phenomenon occurred when the plate and free stream move in the like direction with the plate velocity faster than the free stream velocity or for stationary plate.

Figure. 3 (b) reveals that the dimensionless temperature reduces with the elevating of the magnetic field parameter in case of stationary 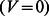 permeable 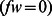 flat plate for uniform free stream 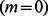. Physically this is because magnetic field enhances the flow velocity, thermal energy is replaced by kinetic energy and this causes reduction of the fluid temperature through the boundary layer. It is further revealed that the dimensionless temperature enhances with the elevating of the magnetic field parameter for the plate which moves in the like direction of the free stream with higher velocity than free stream 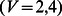. Physically this is due to the fact that as 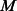
increases the Lorentz force opposes the flow which increases the friction between the fluid layers and hence enhances the temperature.

It is noticed from Fig. 4 (a), that both the Schmidt number and the velocity ratio parameters reduce the concentration both for stationary and moving plates. The plate is moving either in the same or in the opposite direction to the free stream velocity. This is because as the Schmidt number Sc increases due to a reduction in the molecular diffusivity of chemical species for a fixed value of kinematic viscosity, the thickness of the concentration boundary layer shrinks. Figure 4 (b) shows the variations of the non-dimensional concentration with the suction/injection and magnetic field parameters. Like, the temperature, concentration is found to be decreased with the magnetic field parameter for stationary (permeable/solid) plate. As usual, suction increases whilst injection decreases the concentration.

Figure 5 shows the effect of the magnetic field, power law index and suction/injection parameters on the dimensionless friction factor when the plate is either stationary or moves along positive *x*-axis. An inspection of this Fig. reveals that friction factor increases with magnetic filed, index parameter as well as with mass transfer velocity. This trend is true for both the stationary and moving plates. A close observation of Fig. 5 a and 5 b show that friction factor for stationary plate is higher than the moving plate.

The effect of the convective heat transfer, radiation, mass transfer velocity, velocity ratio, magnetic field and Prandtl number on the dimensionless heat transfer rates is exhibited in Fig. 5. It is noticed that the rate of heat transfer increases with the convective heat transfer parameter for both the permeability and solid plate (Fig. 6 (a)). It is further noticed that the heat transfer rate rises with the transpiration velocity. The thermal radiation leads to a reduction of the heat transfer rate for both permeable and impermeable convectively heated plates. Figure 6 (b) reveals that the rate of heat transfer is a monotonic increasing function of the velocity ratio and Prandtl number for both the magnetohydrodynamic and purely hydrodynamic boundary layer flow. It is further seen that the heat transfer rate is an increasing function of the magnetic field parameter for both stationary and moving permeable radiating plate. Finally, Fig. 7 exhibits the effect of the Schmidt number, mass transfer velocity, magnetic field, velocity ratio and power law index parameters on the dimensionless mass transfer rates. From Fig. 7 (a), it is observed that dimensionless the mass transfer rate enhances with Schmidt number for both permeable and solid plate in case of both magnetohydrodynamic and purely hydrodynamic boundary layer flow. The rate of mass transfer is suppressed with the increasing value of the magnetic field whilst it increases for increasing of the velocity ratio and power law index parameters.

## Conclusions

In this paper we studied the problem of heat and mass transfer along a moving permeable radiating vertical flat plate in the moving free stream with the convective surface boundary condition numerically. Similarity transformations have been developed by a linear group of transformation to transform the transport equations into a corresponding system of nonlinear ordinary differential equations. These equations are then solved numerically using the Runge-Kutta-Fehlberg fourth-fifth order numerical method under Maple 13. The main results can be summarized as below:

- The velocity decreases with pressure gradient, mass transfer velocity and the magnetic field parameter.
- Thermal radiation leads to increase temperature and decrease heat transfer rates.
- The temperature, concentration and mass transfer rates decrease with the magnetic field.
- The rate of heat transfer elevates with mass transfer velocity, convective heat transfer, Prandtl number, velocity ratio and magnetic field parameters.
- The rate of mass transfer enhances with the mass transfer velocity, velocity ratio, power law index and the Schmidt number, whilst it falls as the magnetic field increases.
